# *Brachylaima* spp. (Trematoda) parasitizing *Cornu aspersum* (Gastropoda) in France with potential risk of human consumption

**DOI:** 10.1051/parasite/2020012

**Published:** 2020-03-13

**Authors:** Claudia Gérard, Armelle Ansart, Nolwenn Decanter, Marie-Claire Martin, Maxime Dahirel

**Affiliations:** 1 Université de Rennes, CNRS, ECOBIO (Ecosystèmes, biodiversité, Évolution) – UMR 6553 35000 Rennes France; 2 INRAE, Université Côte d’Azur, CNRS, ISA (Institut Sophia Agrobiotech) 06903 Sophia-Antipolis France

**Keywords:** *Cornu aspersum*, *Brachylaima*, Prevalence, Edible land snail, Trematode, Human parasitosis

## Abstract

The edible land snail *Cornu aspersum*, native to the Mediterranean coastlines of North Africa, is widely distributed on most continents and often invasive in areas where introduction is recent. This species could contribute to the geographic spread of parasites as demonstrated for *Brachylaima* spp. These cosmopolitan trematodes may represent a threat to human health, like in Australia where *Brachylaima cribbi* infects humans. In this study, we demonstrate for the first time the occurrence of *Brachylaima* spp. in two French populations of *C. aspersum*, Thorigné-Fouillard (Ille-et-Vilaine), and Arçais (Deux-Sèvres), with an overall prevalence of 10.4% (Thorigné-Fouillard) and 73.3% (Arçais), respectively and a metacercarial intensity on average three times higher in Thorigné-Fouillard (37) than in Arçais (11). *Cornu aspersum* may act as a first and second intermediate host, as demonstrated in Arçais. The morphometrics of metacercariae, particularly the great body length about 2 mm, discriminate our *Brachylaima* species from those already described in *C. aspersum* (*B. cribbi* in Australia, and *B. aspersae*, *B. llobregatensis* and *B. mascomai* in Europe). Molecular analysis, based on 28S and COI, suggests the occurrence of two species in our study, one of which is probably *Brachylaima mesostoma*, an intestinal parasite of passeriform birds described in Central Europe. We underline the need for further research to identify species of *Brachylaima* in France and measure the health hazard of consuming field-collected snails.

## Abstract (fr)

L’escargot *Cornu aspersum*, originaire des côtes méditerranéennes d’Afrique du Nord, est largement répandu sur la plupart des continents et souvent invasif dans ses aires d’introduction récente. Cette espèce peut contribuer à l’expansion géographique de parasites comme démontré pour *Brachylaima* spp. Ces trématodes cosmopolites peuvent représenter une menace pour la santé comme en Australie où *Brachylaima cribbi* parasite l’espèce humaine. Dans cette étude, nous démontrons pour la première fois la présence de *Brachylaima* spp. dans deux populations françaises de *C. aspersum*, à Thorigné-Fouillard (Ille-et-Vilaine) et Arçais (Deux-Sèvres), avec une prévalence totale de 10.4 % (Thorigné-Fouillard) et 73.3 % (Arçais), et une intensité de métacercaires en moyenne trois fois plus importante à Thorigné-Fouillard (37) qu’à Arçais (11). *Cornu aspersum* peut jouer le rôle de premier et de second hôte intermédiaire, comme démontré à Arçais. La morphométrie des métacercaires, en particulier la grande taille corporelle d’environ 2 mm de long, suggèrent qu’il ne s’agit pas d’espèces de *Brachylaima* déjà décrites chez *C. aspersum* (*B. cribbi* en Australie et *B. aspersae*, *B. llobregatensis* et *B. mascomai* en Europe). L’analyse moléculaire, basée sur 28S et COI, suggère la présence de deux espèces dans notre étude, dont l’une est probablement *Brachylaima mesostoma*, un parasite intestinal d’oiseaux passeriformes décrit en Europe Centrale. Nous soulignons le besoin de recherche future pour identifier les espèces de *Brachylaima* présentes en France et mesurer le risque associé à la consommation d’escargots terrestres prélevés en milieu naturel.

## Introduction

The land snail *Cornu aspersum aspersum* Müller (syn. *Helix aspersa*) (Gastropoda), native to the Mediterranean coastlines of North Africa, is now widely distributed, occurring on all continents except Antarctica, as well as on numerous islands [1, 20, 21]. *Cornu aspersum* is particularly abundant in human-disturbed habitats under favorable climatic conditions (Mediterranean temperate and subtropical) and is considered an invasive and pest species in many regions of its introduced areas, such as in the Americas and Oceania [1, 20]. Its successful spread is at least partly explained by inadvertent and intentional human introductions, but also by high phenotypic plasticity resulting in various adaptive morpho-anatomic, physiological, and behavioral responses to environmental fluctuations (e.g., [14, 29, 39, 41]).

When invasive species serve as hosts, they can strongly influence infectious disease dynamics in invaded areas (for reviews: [12, 46, 47]). It is therefore crucial to know the parasitofauna recorded in invasive host species in order to understand or predict the possible impacts of their parasites. Helminth parasites of *C. aspersum* are mostly Nematoda (at least 16 species including four facultative parasite species) (Table 1), and to a lesser extent Trematoda (*Dicrocoelium dendriticum* and four species of *Brachylaima*) (Table 2). Some authors [15] clearly demonstrated that imported edible *C. aspersum* specimens could contribute significantly to the geographic spread of *Brachylaima* species, in their case between Spain and Africa. *Cornu aspersum* also plays a key role in the transmission of emerging helminthiases of veterinary importance such as feline aelurostrongylosis and canine angiostrongylosis [9, 10]. Moreover, some species parasitizing *C. aspersum* at the larval stage such as *Angiostrongylus cantonensis* and *Brachylaima cribbi* can infect humans as definitive hosts with a lethal risk (>10% and 5–10% mortality rate without treatment, respectively), and thus, represent a serious human health hazard [13, 31, 55]. More generally, species of the genus *Brachylaima* may have both veterinary and medical significance since they can occur in various domestic birds and mammals, including poultry, pigeons, pigs, rabbits, and dogs, representing potential reservoirs for human infection [22, 34, 43, 57].

**Table 1:** Nematode species parasitizing *Cornu aspersum* as intermediate or definitive hosts.

Parasite species	References
*Cornu aspersum* as intermediate host	
Order Strongylida	
*Aelurostrongylus abstrusus* (Railliet, 1898)	[9]
*Angiostrongylus cantonensis* Chen, 1935	[7]
*Angiostrongylus dujardini* Drozdz & Doby, 1970	[19]
*Angiostrongylus vasorum* (Baillet, 1866)	[10]
*Crenosoma vulpis* (Dujardin, 1844)	[8]
*Morerastrongylus andersoni* (Petter, 1972)	[44]
*Neostrongylus linearis* (Marotel, 1913)	[51]
*Oslerus rostratus* Gerichter, 1945	[19]
*Protostrongylus rufescens* (Leuckart, 1865)	[19]
*Troglostrongylus brevior* Gerichter, 1948	[7]
Order: Rhabditida (facultative parasites)	
*Alloionema appendiculata* Schneider, 1859	[35]
*Caenorhabditis elegans* Maupas, 1900	[49]
*Phasmarhabditis hermaphrodita* Schneider, 1859	[35]
*Rhabditis gracilicaudata* de Man, 1876	[35]
*Cornu aspersum* as definitive host	
Order: Ascaridida	
*Nemhelix bakeri* Morand & Petter, 1986	[37]
Order: Rhabditida	
*Angiostoma aspersae* Morand, 1986	[36]

**Table 2:** Digenean trematode species parasitizing *Cornu aspersum* as first (Hi1) and/or second (Hi2) intermediate host.

Parasite species	Larval stages in Hi (MH)	Definitive host	Country	References
Plagiorchiida				
*Dicrocoelium dendriticum* (Rudolphi, 1819)	SP/C in Hi1 (DG)	Sheep, goats	Turkey	[23, 26]
Diplostomida				
*Brachylaima* sp. Dujardin, 1843	SP/C in Hi1 (DG)		Turkey	[26]
*Brachylaima aspersae* Segade et al., 2011	SP/C in Hi1 (DG); MC in Hi2 (K)	Rodents	Spain	[53]
*Brachylaima cribbi* Butcher & Grove, 2001	MC in Hi2 (K)	Mammals, birds*, reptiles	Australia	[3, 4]
*Brachylaima llobregatensis* González-Moreno & Gracenea, 2006	SP/C in Hi1 (DG); MC in Hi2 (K)	Rodents	Algeria, Spain	[15, 16]
*Brachylaima mascomai* Gracenea & González-Moreno, 2002	MC in Hi2 (K)	Rodents	South Africa, Spain	[15, 18]

Larval stages: SP, sporocysts; C, cercariae; MC, metacercariae. MH, Microhabitat (MH) in parenthesis: DG, digestive gland; K, kidney.

* Introduced European *Turdus merula* and *Sturnus vulgaris* being the most commonly infected natural definitive hosts.

Up to now, *B. cribbi* is the first brachylaimid trematode known to infect humans and is recorded exclusively in Australia [31]. Human brachylaimiasis (intestinal fluke infection) occurs after consumption of undercooked land snails including *C. aspersum* parasitized by infective metacercariae [3], or also by viable metacercariae deposited on vegetables via the snail’s slime trail and excreta and/or crushed snails [6]. Hematophagous adults of *B. cribbi* inhabit the intestine, and their eggs are recovered in human feces [43]. *Brachylaima cribbi* is thought to be of European origin: most of its intermediate hosts are helicid species introduced to Australia from Europe, and the introduced European birds *Turdus merula* (common blackbird) and *Sturnus vulgaris* (common starling) are its most commonly infected natural definitive hosts among mammals, birds, and reptiles [3, 4].

Three other species of the cosmopolitan genus *Brachylaima* parasitize *C. aspersum* in other countries apart from Australia, all involving rodents as definitive hosts (Table 2). *Brachylaima aspersae* and *Brachylaima llobregatensis* use *C. aspersum* as first and second intermediate hosts in Spain, and also in Algeria for *B. llobregatensis* [15, 53]. *Brachylaima mascomai*, for which *C. aspersum* is one of the second intermediate host species, occurs in Spain and in South Africa [15]. An undetermined species of *Brachylaima* was also recorded in *C. aspersum* from Turkey, acting as first intermediate host [26]. In Europe, no epidemiological data are available up to now, except in Spain where the prevalence of *Brachylaima* spp. metacercariae in *C. aspersum* from marketplaces varies from 0% to 93.6% depending on the season and region [17]. It is worth highlighting the increasing worldwide interest in *Brachylaima* spp. infecting other terrestrial snail species, according to recent studies (e.g., [59, 60]). In France, a country bordering Spain, people consume between 25,000 and 30,000 tons of edible land snails per year, among them 800–1000 tons of *C. aspersum* produced on farms [32]. Moreover, *C. aspersum* is an anthropophilous species, quite common in urban areas and private gardens [25]. Due to the potential risk of brachylaimiasis for humans and domestic animals related to the consumption of infected land snails and/or vegetables with viable metacercariae, the main objective of our preliminary study was to investigate the occurrence of *Brachylaima* spp. in two allopatric populations of *C. aspersum* in France. If present, we also aimed (i) to research whether *C. aspersum* can act as first and/or second intermediate host, and (ii) to provide some morphological and molecular data for further comparison with *Brachylaima* species already described.

## Materials and methods

### Study sites, sampling, and measurements

We sampled two wild populations of *C. aspersum* in northwestern France, distant from each other by about 220 km as the crow flies. In total, 326 snails were collected at the end of hibernation/beginning of physiological awakening: 49 specimens from Thorigné-Fouillard (Department Ille-et-Vilaine, Region Bretagne; 48°15′51″ N, 1°57′74″ W) on 18 February 2018, and 277 specimens from Arçais (Department Deux-Sèvres, Region Nouvelle-Aquitaine; 46°17′47.9″ N, 0°41′32.6″ W) on 1 April 2018. Both populations inhabit private gardens, but in a suburban area for Thorigné-Fouillard, whereas rural for Arçais.

We distinguished adults from subadults by the presence of a reflected shell lip indicating the cessation of shell growth [1]. We froze all the snails prior to the search for larval trematodes in various organs (lungs, heart, kidney, body cavity, and digestive gland), and we dissected them using a binocular microscope. We observed trematodes (sporocysts, cercariae, and/or metacercariae), when present, under light glass coverslip pressure using bright-field and phase-contrast microscopy. We counted and morphologically identified sporocysts and metacercariae of *Brachylaima* spp. according to Gracenea and González-Moreno [18] and Segade et al. [53]. We performed measurements of metacercariae preserved in 95% Ethanol, following Mas-Coma et al. [30]. Sporocysts and metacercariae of *Brachylaima* spp. were also preserved in 95% ethanol for DNA sequence analysis (see below).

We described parasitism by prevalence (P%) (number of hosts infected with a particular parasite species/number of examined hosts), mean abundance (A) (average abundance of a parasite species among all members of a host sample), and mean intensity (*I*) (total number of parasites of a particular species found in a sample/number of hosts infected with that parasite) [2].

### DNA sequencing of *Brachylaima* spp., alignment, and phylogenetic analyses

We analyzed 13 sporocysts and 9 metacercariae of *Brachylaima* spp., each of them originating from a different snail, by molecular identification tools. DNA of each individual parasite (approximately 1 mm^3^ piece of sporocyst or whole body of a metacercaria) was extracted following [38]. Samples were lysed in 25 μL of 0.02 N NaOH at 99 °C for 30 min. We amplified fragments of ca. 1275 and 780 bp (except one shorter sequence of 669 bp) for the nuclear 28S ribosomal DNA (rDNA) and mitochondrial COI DNA (mtDNA) genes, respectively. The 28S fragment was amplified using the forward primer dig12 (5′ – AAGCATATCACTAAGCGG – 3′) and the reverse primer 1500R (5′ – GCTATCCTGAGGGAAACTTCG – 3′) [58]. The COI region was amplified using the forward primer JB3 (5′ – TTTTTTGGGCATCCTGAGGTTTAT – 3′) and the reverse primer CO1-R trema (5′ – CAACAAATCATGATGCAAAAGG – 3′) [33]. Amplification of template DNA was carried out in 12 μL volumes with MyTaq Mix (2X) (Bioline, France), including 5 μM of each primer and 2 μL of DNA. The PCR conditions were 98 °C for 10 s, 50 °C for 20 s, and 68 °C for 90 s (40 cycles). We checked amplification products using a 2% agarose gel stained with ethidium bromide. We obtained double-strand sequences with an automated sequencer (Plateforme de séquençage et génotypage OUEST-genopole^®^). New 28S and COI sequences were submitted to GenBank (Table 4).

We retrieved comparative sequences of related taxa from the superfamily Brachylaimoidea from GenBank databases, as well as sequences of *Clinostomum* species from the superfamily Schistosomatoidea, used as the outgroup. We discarded sequences that were too short or aligned too ambiguously with our dataset. Nuclear 28S sequences generated in this study were aligned using the built-in assembly algorithm implemented in CODONCODE ALIGNER software v8.0.1 (CodonCode Corporation, Dedham, MA, USA), whereas mitochondrial COI sequences were aligned using the on-line algorithm for coding sequences MACSE (Multiple Alignment of Coding Sequences, accounting for frameshifts and stop codons) [50].

We estimated phylogenetic relationships between our samples and sequences retrieved from GenBank by Bayesian inference (BI). We selected the best model of nucleotide substitutions prior to BI analyses using the Akaike information criterion (AIC). We used MRAIC software, v1.4.2 [42] to evaluate 24 different models of nucleotide substitutions. The resulting best models were GTR + G for 28S rDNA and HKY + G for COI mtDNA (GTR: generalized time reversible; HKY: Hasegawa–Kishino–Yano). We incorporated these models in MRBAYES v3.1.1-p1 [52] for BI analyses. We conducted phylogenetic inference independently for each target gene, but also for both genes simultaneously, concatenated into a super-gene alignment of 2102 bp, although only for species and samples for which COI and 28S sequences were available. We approximated the posterior probabilities of trees and parameters with Markov Chain Monte Carlo (MCMC) and Metropolis coupling. We ran two independent MCMC analyses with four chains each and a temperature set to 0.2. Each chain was run for 10,000,000 cycles with trees sampled every 100 generations. Posterior probabilities were obtained from the 50% majority rules consensus of trees sampled after discarding the trees saved before chains reached apparent stationarity (i.e., a “burn-in period” of 8000 generations). For each analysis, the average standard deviation of split frequencies after 10,000,000 generations was well below 0.01, indicating very good convergence between the two runs.

### Statistical analysis

We performed the analyses using R, version 3.5.1 [48]. We used Bernoulli generalized linear models (GLMs) to analyze the prevalence of metacercariae and sporocysts (binary variables: infected/uninfected). We tested whether infection probability depended on site, life stage, and their interaction. We undertook the same analysis on metacercariae counts, using this time a quasi-Poisson GLM (due to evidence of overdispersion). When a variable had a significant effect, post-hoc comparisons among groups were performed using estimated marginal means and contrasts [27]. Due to the low number of positive cases, data for sporocyst counts were not analyzed statistically but simply presented (Table 3).

**Table 3:** Occurrence of *Brachylaima* sporocysts (SP) and metacercariae (MC) in *C. aspersum* from Arçais and Thorigné-Fouillard.

	Shell size	P% SP	A SP	I SP	P% MC	A MC	I MC
Arçais							
Subadults (139)	23.93 ± 0.36	5.8 (2.9–10.9)	3.50 ± 2.89	69.43 ± 35.93	72.7 (64.7–79.4)	9.21 ± 2.12	12.67 ± 2.65
Adults (138)	30.53 ± 0.30	3.6 (1.6–8.2)	1.57 ± 1.78	51.40 ± 55.49	68.8 (60.7–76.0)	6.05 ± 1.61	8.79 ± 2.15
All snails (277)	27.24 ± 0.46	4.7 (2.8–7.9)	2.68 ± 1.79	61.92 ± 26.79	70.8 (65.2–75.8)	7.64 ± 1.34	10.79 ± 1.71
Thorigné-Fouillard							
Subadults (36)	19.54 ± 0.95	–	–	–	2.8 (0.5–14.2)	0.72 ± 1.47	26
Adults (13)	29.25 ± 1.21	–	–	–	38.5 (17.7–64.5)	14.92 ± 21.31	38.80 ± 59.05
All snails (49)	22.12 ± 1.47	–	–	–	12.2 (5.7–24.2)	4.49 ± 5.55	36.67 ± 46.18

P%: prevalence, A: abundance, I: intensity. All values are means with 95% CI in parentheses.

Differences were considered statistically significant at *p* ≤ 0.05. We reported mean values followed by the 95% confidence interval (CI). For prevalence, we calculated CI using the score method [40].

## Results

### Occurrence of *Brachylaima* spp. in *Cornu aspersum* from Arçais and Thorigné-Fouillard

**Figure 1:**
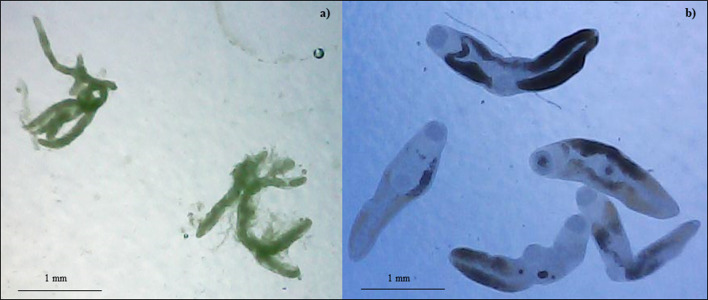
(a) Sporocysts and (b) metacercariae of *Brachylaima* found in *Cornu aspersum* at sites in France. Sporocysts were only recorded in Arçais, whereas metacercariae occurred in Arçais and Thorigné-Fouillard.

Land snails from both sites were infected by *Brachylaima* spp. with a total prevalence of 73.3% (CI [67.8%–78.2%]) and 12.2% (CI [5.7%–24.2%]) in Arçais and Thorigné-Fouillard, respectively (Table 3, Fig. 1). No other digenean trematodes besides *Brachylaima* spp. were present. In Arçais, *C. aspersum* mainly plays the role of second intermediate host for *Brachylaima* spp. (196 among 277 snails) and harbors up to 100 metacercariae in the kidney (Table 3). More rarely, *C. aspersum* acts as first intermediate host (13 among 277 snails), harboring up to 100 sporocysts in the digestive gland (Table 3). In total, six snails were concomitantly infected by sporocysts and metacercariae. In Thorigné-Fouillard, no individuals of *C. aspersum* were infected by sporocysts (prevalence of 0.0%, CI [0.0–7.3%]). *Cornu aspersum* was found to play only the role of second intermediate host for *Brachylaima* spp. (5 among 49 snails) and harbored up to 100 metacercariae in the kidney (Table 3). One adult snail from Thorigné-Fouillard harbored metacercariae both in the kidney (63) and in the lung cavity (45).

### Molecular analysis and morphometrics of *Brachylaima* spp. in *Cornu aspersum*

**Figure 2:**
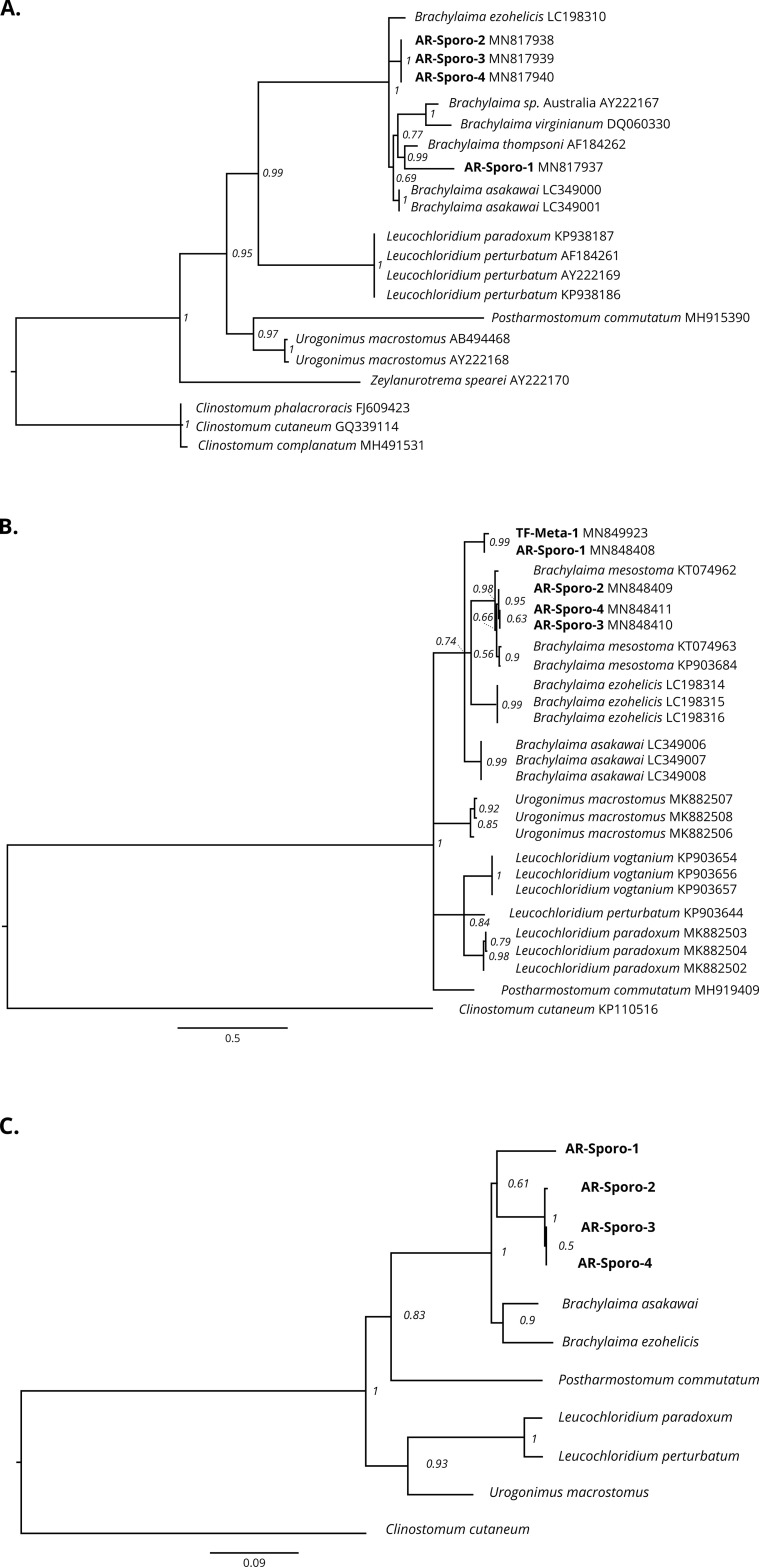
Fifty percent majority-rule consensus phylogram from the Bayesian inference (BI) analysis of (A) 28S rDNA, (B) mtDNA COI, and (C) both concatenated sequences of unidentified trematode specimens from two *Cornu aspersum* populations (in bold) and related species from the Brachylaimidae and Leucochloridiidae families. The trees are rooted using *Clinostomum* sp. (Family Clinostomidae) as the outgroup. Posterior probability values (in italics) are indicated near the branch nodes. Scale bars indicate the number of substitutions per nucleotide site. See Table 4 for information on taxonomy and sample origin, and Supplementary Tables 1–3 for information on the percentage of identity among sequences.

We obtained good quality 28S rDNA and COI mtDNA sequences for four sporocysts from Arçais, and only one COI mtDNA sequence for a metacercaria from Thorigné-Fouillard. Unfortunately, DNA isolation was unsuccessful for the other samples of *Brachylaima* spp. sporocysts and metacercariae used for molecular analysis. The successful sequences were implemented in the phylogenetic analyses (Table 4; sequence identity matrices are presented in Supplementary Tables 1–3). Phylogenetic trees illustrating the relationships between our samples and related species from GenBank were globally well supported, with posterior probability values never below 0.5 (Fig. 2). The monophyly of the genus *Brachylaima* was very well supported (probability posterior value ≥ 0.67), and our larval trematodes were unambiguously identified as *Brachylaima* species (Fig. 2).

**Table 4:** Taxonomy and GenBank accession number of the 28S and COI sequences for the species related to our study samples and used in subsequent phylogenetic analyses.

Taxon	Stage	Host species	Country	28S	COI
Brachylaimoidea					
Brachylaimidae					
*Brachylaima asakawai*	A	*Myodes rufocanus*	Japan	**LC349000**	**LC349006**
	M	*Discus pauper*	Japan	LC349001	LC349007
	M	*Succinea lauta*	Japan	–	LC349008
*Brachylaima ezohelicis*	M	*Ezohelix gainesi*	Japan	**LC198310**	LC198314
	M	Idem	Japan	–	LC198315
	M	Idem	Japan	–	LC198316
*Brachylaima mesostoma*	A	*Turdus philomelos*	Czech R	–	KP903684
	A	*Sylvia atricapilla*	Poland	–	KT074962
	A	Idem	Poland	–	KT074963
*Brachylaima sp Australia*	A	*Mus musculus*	Australia	AY222167	–
*Brachylaima thompsoni*	A	*Blarina brevicauda*	USA	AF184262	–
*Brachylaima virginianum*	A	*Didelphis virginiana*	USA	DQ060330	–
*Postharmostomum commutatum*	A	*Gallus gallus*	Brazil	**MH915390**	**MH919409**
*Zeylanurotrema speraei*	A	*Rhinella marina*	Australia	AY222170	–
Leucochloridiidae					
*Leucochloridium paradoxum*	S	*Succinea sp.*	Russia	**KP938187**	**MK882502**
	A	*Parus major*	Baltic coast	–	MK882503
		*Cyanistes caeruleus*			
	A	Idem	Baltic coast	–	MK882504
*Leucochloridium perturbatum*	A	*Turdus philomelos*	Czech R	**KP938186**	**KP903644**
	A	*Turdus merula*	Czech R	AY222169	–
	S	Terrestrial snail	Poland	AF184261	–
*Leucochloridium vogtianum*	A	*Acrocephalus arundinaceus*	Czech R	–	KP903667
	A	*Locustella fluviatilis*	Czech R	–	KP903656
	A	*Acrocephalus arundinaceus*	Czech R	–	KP903654
*Urogonimus macrostomus*	A	*Parus major*	Baltic coast	**AY222168**	MK882506
		*Cyanistes caeruleus*			
	A	Idem	Baltic coast	–	MK882508
	A	*Emberiza rustica*	Japan	AB494468	**MK882507**
Schistosomatoidea					
Clinostomidae					
*Clinostomum complanatum* *	A	*Phalacrocorax carbo*	Japan	MH491531	–
*Clinostomum cutaneum* *	A	*Ardea cinerea*	Kenya	**GQ339114**	**KP110516**
*Clinostomum phalacrocoracis* *	A	Idem	Kenya	FJ609423	–
Sequences in this study					
AR-Sporo-1	S	*Cornu aspersum*	Arçais	**MN817937**	**MN848408**
AR-Sporo-2	S	Idem	Arçais	**MN817938**	**MN848409**
AR-Sporo-3	S	Idem	Arçais	**MN817939**	**MN848410**
AR-Sporo-4	S	Idem	Arçais	**MN817940**	**MN848411**
TF-Meta-1	M	Idem	Thorigné-F.	–	**MN849923**

Parasitic stage (A: adult, M: metacercaria, S: sporocyst), host species and country are indicated. Sequences in bold are those used for the two-gene BI analysis.

* Outgroup.

Three samples from Arçais (AR-Sporo-2, AR-Sporo-3, and AR-Sporo-4) form a clade that could be *Brachylaima mesostoma* according to the COI tree (Fig. 2B). Unfortunately, 28S rDNA sequences available in GenBank for *B. mesostoma* were too short and could not be included in the analyses.

The other Arçais sporocyst sample (AR-Sporo-1) did not branch with the three others inside the *Brachylaima* clade, but with the metacercarial Thorigné-Fouillard sample (TF-Meta-1) based on the COI tree (Fig. 2B) and concatenated genes tree (Fig. 2C). This suggests that at least two *Brachylaima* species occur in Arçais, potentially corresponding to *B. mesostoma* and an unidentified species also occurring in Thorigné-Fouillard.

The morphometrics of *Brachylaima* metacercariae were similar in Arçais and Thorigné-Fouillard, but were of greater size than those of *B. mesostoma* coming from the hygromiid *Helicopsis retowskii* (Table 5). Metacercariae from both sites were also of greater size than those of *B. aspersae*, *B. cribbi*, *B. llobregatensis*, and *B. mascomai* known to use *C. aspersum* as second intermediate host (Table 5). No DNA sequences are available in GenBank for these four species infecting *C. aspersum*.

**Table 5:** Measurements (in μm) of *Brachylaima* metacercariae in *Cornu aspersum* from Thorigné-Fouillard (*N* = 10) and Arçais (*N* = 10), and comparison with the four *Brachylaima* species recorded in *C. aspersum* (*B. aspersae*, *B. cribbi, B. llobregatensis*, and *B. mascomai*) and with *Brachylaima mesostoma*. Measurements of *B. aspersae* and *B. cribbi* are reported from metacercariae found in *C. aspersum* [3, 53], those of *B. llobregatensis* and *B. mascomai* from the helicid *Otala punctata* [18, 42], and those of *B. mesostoma* from the hygromiid *Helicopsis retowskii* [57].

	In *C. aspersum*								In *O. punctata*				In *H. retowskii*	
	Thorigné (*N* = 3)		Arçais (*N* = 4)		*B. aspersae*		*B. cribbi*		*B. llobregatensis*		*B. mascomai*		*B. mesostoma*	
	Mean	Range	Mean	Range	Mean	Range	Mean	Range	Mean	Range	Mean	Range	Mean	Range
Body														
Length	2046	2000–2126	2005	1832–2179	644	495–882	1152	625–1912	1243	982–1485	1321	604–1814	1323	896–1566
Width	498	442–547	584	558–611	385	113–470	410	183–608	407	822–1485	419	248–648	512	400–648
Oral sucker														
Length	225	211–232	289	274–316	156	130–180	174	113–223	195	105–299	196	105–299	226	190–259
Width	263	263	268	253–284	150	125–173	163	108–203	179	137–235	182	100–235	223	175–265
Ventral sucker														
Length	263	253–274	237	221–253	140	113–163	137	100–180	169	145–235	173	132–235	174	148–212
Width	249	221–263	234	221–253	126	103–158	153	103–198	159	131–224	162	101–224	190	162–217
Pharynx														
Length	144	137–147	134	105–158	96	80–113	81	54–113	87	66–132	88	66–132	123	101–132
Width	172	147–189	129	126–137	100	30–190	97	60–140	120	92–158	121	92–158	122	111–132

### Comparison of *Brachylaima* spp. in *Cornu aspersum* between sites and life stages

The metacercarial prevalence in Arçais was six times higher than in Thorigné-Fouillard (analysis of deviance, $\chi_{1}^{2}$ = 59.73, *p* < 0.0001), with a significant stage-by-site interaction ($\chi_{1}^{2}$ = 10.42, *p* = 0.001), whereas the intensity of metacercariae was more than three times lower (Table 3). The metacercarial abundance depended on both site and life stage (interaction effect, $\chi_{1}^{2}$ = 22.45, *p* < 0.0001).

In Arçais, no significant differences between subadults and adults were detected in the prevalence and the abundance of metacercariae (contrast of marginal means: *p* = 0.90 and *p* = 0.15, respectively). In Thorigné-Fouillard, both prevalence (contrast *p* = 0.04) and abundance of metacercariae (*p* = 0.007) were higher in adults than in subadults (Table 3).

There was no detectable effect of site or life stage on sporocyst prevalence (all *p* > 0.06).

## Discussion

Species of the digenean trematode *Brachylaima* were recorded for the first time in populations of *C. aspersum* sampled in France. Two other land snail species, the helicids *Cepaea nemoralis* and *Cepaea hortensis*, were previously found to harbor *Brachylaima* metacercariae at another site in France (i.e., Richelieu located at 110 km from Arçais and 190 km from Thorigné-Fouillard as the crow flies), but without description of the parasite species [11]. In Poland, a recent study described the occurrence of *Brachylaima mesostoma* cercariae and metacercariae in three among 11 populations of *C. nemoralis* and *C. hortensis*, with spring prevalence up to 54% and 60%, respectively [60]. In our study, *C. aspersum* was found to act as both first and second intermediate host in Arçais, whereas only as second in Thorigné-Fouillard. The absence of *C. aspersum* infected by sporocysts in Thorigné-Fouillard may be due to the low sampling effort. In fact, prevalence of *Brachylaima* sporocysts was low in Arçais (≤5%), as generally recorded in *C. aspersum* from Spain for *B. aspersae* [53] and *B. llobregatensis* [16], and from Turkey for an undetermined *Brachylaima* species [26].

The prevalence of *Brachylaima* metacercariae in Thorigné-Fouillard (12%) was about six times lower than in Arçais (71%). This may be partly explained by differences in the sampling date, respectively mid-February (end of winter when snails were just coming out of hibernation) for Thorigné-Fouillard vs. 1 April (beginning of spring with fully active snails) for Arçais. Seasonal fluctuations occur in the metacercarial prevalence of *Brachylaima* spp. in Spain, with the lowest value recorded in winter (23%) [17].

Another explanation of higher metacercarial prevalence in Arçais may be the higher density of *C. aspersum* and other land snail species potentially acting as second intermediate host (i.e., *C. nemoralis* and the hygromiids *Cernuella virgata*, *Cochlicella acuta*, *Theba pisana*, and *Trochoidea elegans*) than in Thorigné-Fouillard (A. Ansart, pers. obs.). This higher density favors contact between snails, and consequently, *Brachylaima* spp. transmission from first to second intermediate hosts [15]. Importantly, cercariae of *Brachylaima* emerging from sporocyst-infected snails crawl actively on humid substrate until they come into contact with a susceptible second intermediate snail host [17]. A wide specificity of *Brachylaima* spp. generally occurs for the second intermediate host as for *B. cribbi* metacercariae infecting various helicid and hygromiid species in Australia [5]. Contrastingly, the specificity of *Brachylaima* spp. is clearly more restricted for the first intermediate host, and even, is oioxenic for *B. aspersae* and *B. llobregatensis* parasitizing *C. aspersum* [16, 53]. According to the life cycle of *B. aspersae* [53], after egg ingestion by *C. aspersum*, the miracidium hatches and develops into a highly branched sporocyst in the digestive gland. Cercariae produced by sporocysts emerge from the first intermediate host and enter the kidney of the second via the ureter. Then, they feed on the renal epithelium to develop into nonencysted fully mature metacercariae. Cercariae are unable to infect the snail from which they are emerging; therefore, autoinfection is not possible [53]. Despite this, *C. aspersum* can be infected simultaneously with sporocysts and metacercariae of *B. aspersae* [53].

Differences in populations of definitive hosts between sites may induce some differences in prevalence of *Brachylaima* spp. in first and second intermediate hosts. Rodents are the main definitive hosts of *Brachylaima* spp. recorded in *C. aspersum* from Europe, i.e. *B. aspersae*, *B. llobregatensis*, and *B. mascomai* [16, 18, 53]. Populations of rodents are likely different between suburban (Thorigné-Fouillard) and rural (Arçais) sites [28], with consequences on the probability of ingesting *Brachylaima* spp. eggs released in rodent feces by *C. aspersum* used as first intermediate hosts, potentially inducing inter-site differences in parasite prevalence. Birds are also important definitive hosts of *Brachylaima* spp. as demonstrated for *B. cribbi* commonly infecting introduced European turdids (*T. merula* and *S. vulgaris*) in Australia [3, 4], as well as for *B. mesostoma*, *Brachylaima arcuatus*, and *Brachylaima fuscata* infecting passerines (*Sylvia atricapilla*, *Turdus philomelos*, *T. merula*, *Garrulus glandarius*) in Central Europe (Poland, Czech Republic) [24]. As for rodents, passerine diversity and abundance probably differ between rural and suburban settings [56], potentially influencing *Brachylaima* spp. prevalences.

The three time-higher intensity of metacercariae in *C. aspersum* from Thorigné-Fouillard compared to Arçais may reveal a different strategy of *Brachylaima* spp. depending on the site, with a higher degree of parasite aggregation in the snail population of Thorigné-Fouillard (lower prevalence with higher intensity). In Arçais, the distribution of sporocysts was strongly aggregated in the *C. aspersum* population with low prevalence (4%) and high mean parasite intensity, about six times higher than for metacercariae present in more than 70% of the snails. Asexual reproduction of sporocysts in their snail host partly explains differences in aggregation degree between sporocysts and metacercariae [45]. Aggregation of parasitic helminths such as trematodes within host populations (i.e., small proportion of hosts infected with many parasites) is a general law of parasite ecology [45, 54]. The aggregation degree mainly depends on the distribution of hosts and/or infective parasites across space and time, and may be influenced by parasite accumulation with host age [45, 54]. Massive infections with sporocysts or metacercariae of *Brachylaima* can induce extensive pathological effects, i.e. sporocysts can almost totally invade and replace the digestive gland and they can also infiltrate the pulmonary, renal, and gonadal tissues, whereas metacercariae directly feed on the renal epithelium [53]. Thus, one can expect acute harmful effects of *Brachylaima* spp. on the few snails parasitized by sporocysts in Arçais and those parasitized by metacercariae in Thorigné-Fouillard. In contrast, the lower number of *Brachylaima* metacercariae in subadult and adult snails from Arçais may suggest a more efficient immune response against this parasitic larval stage, potentially limiting parasite-induced lethal risk.

Concerning identification of the *Brachylaima* spp. in this study, our preliminary genetic data suggest that at least two different *Brachylaima* species parasitize *C. aspersum* at the two study sites. One of the species, using *C. aspersum* as a first intermediate host in Arçais, is very similar to *B. mesostoma* found in *S. atricapilla* (European blackcap) from Poland and *T. philomelos* (song thrush) from the Czech Republic [24]. The life cycle of *B. mesostoma* was previously described in the Ukraine; there, it does not involve *C. aspersum* but the hygromiid *Xeropicta krynickii* as a first intermediate host and various terrestrial snails (*Xeropicta krynickii*, *Helicopsis retowskii*, *Helicopsis filimargo*, *Eobania vermiculata*, *Brephulopsis cylindrica*, *Brephulopsis bidens*, *Thoanteus gibber*) as second hosts [57]. Recently, *B. mesostoma* was identified in *C. nemoralis* and *C. hortensis* from Poland used both as first and second intermediate hosts [60]. Concerning the second *Brachylaima* species, which is undetermined, both molecular results and similarities in metacercariae morphometrics imply that it may occur at both sites, using *C. aspersum* as first and second intermediate host. Moreover, the great size of metacercariae in Arçais and Thorigné-Fouillard suggests that the *Brachylaima* species here may be different from the four others known to use *C. aspersum*, at least as a second intermediate host (Tables 2 and 5). This hypothesis could be verified via molecular analysis, but unfortunately, no DNA sequences of these four species are available in GenBank allowing comparison with our DNA sequences. Therefore, further studies are needed to specifically identify the *Brachylaima* spp. recorded in populations of *C. aspersum* in France, and to assess the occurrence of *B. mesostoma* and other *Brachylaima* species. It is also important to determine what other land snail species act as intermediate hosts in the field (helicids including *Cepaea* spp. and hygromiids), as well as what species are definitive hosts, potentially including humans and domestic animals. Because global warming provides favorable environmental conditions for the successful spread of *C. aspersum* [14], and therefore the spread of parasites such as *Brachylaima* spp. [15], it is crucial to evaluate the potential risk to human and veterinary health.

## Supplementary materials

Supplementary material is available at https://www.parasite-journal.org/10.1051/parasite/2020012/olm *Supplementary Table 1.* Percentages of identity between the 28S sequences used in the present study. Values were estimated based on aligned sequences using the SeqinR package (Charif and Lobry 2007, DOI: 10.1007/978-3-540-35306-5_10).
*Supplementary Table 2.* Percentages of identity between the COI sequences used in the present study. Values were estimated based on aligned sequences using the SeqinR package (Charif and Lobry 2007, DOI: 10.1007/978-3-540-35306-5_10).
*Supplementary Table 3.* Percentages of identity between the COI + 28S supergene alignment sequences used in the present study. GenBank accession numbers refer respectively to 28S and COI sequences. Values were estimated based on aligned sequences using the SeqinR package (Charif and Lobry 2007, DOI: 10.1007/978-3-540-35306-5_10).
