# Supplementary material for: Brachylaima spp. (Trematoda) parasitizing Cornu aspersum (Gastropoda) in France with potential risk of human consumption
Source: Parasite. 2020 Mar 13;27:15. doi: 10.1051/parasite/2020012 (PMC7069358; doi:10.1051/parasite/2020012)
Supplement: Supplementary file 1 — Supplementary Table 1. Percentages of identity between the 28S sequences used in the present study. Values were estimated based on aligned sequences using the SeqinR package (Charif and Lobry 2007, DOI: 10.1007/978-3-540-35306-5_10). Supplementary Table 2. Percentages of identity between the COI sequences used in the present study. Values were estimated based on aligned sequences using the SeqinR package (Charif and Lobry 2007, DOI: 10.1007/978-3-540-35306-5_10). Supplementary Table 3. Percentages of identity between the COI + 28S supergene alignment sequences used in the present study. GenBank accession numbers refer respectively to 28S and COI sequences. Values were estimated based on aligned sequences using the SeqinR package (Charif and Lobry 2007, DOI: 10.1007/978-3-540-35306-5_10). [file parasite-27-15-s1.pdf]

Supplementary Tables for "*Brachylaima* spp. (Trematoda) parasitizing *Cornu aspersum* (Gastropoda) in France with potential risk for human consumption." Claudia Gérard, Armelle Ansart, Nolwenn Decanter, Marie-Claire Martin and Maxime Dahirel

Supplementary Table 1. Percentages of identity between the 28S sequences used in the present study. Values were estimated based on aligned sequences using the SeqinR package (Charif and Lobry 2007, doi:10.1007/978-3-540-35306-5_10)

Species/sample name	GenBank acc.n°	LC198310	MN817938	MN817939	MN817940	AY222167	DQ060330	AF18262	MN817937	LC349000	LC349001	KP938187	AF184261	AY222169	KP938186	MH915390	AB494468	AY222168	AY222170	FJ609423	GQ339114
Brachylaima ezohelicis	LC198310																				
AR-Sporo-2	MN817938	98.5																			
AR-Sporo-3	MN817939	98.5	100																		
AR-Sporo-4	MN817940	98.5	100	100																	
Brachylaima sp. Australia	AY222167	96.99	96.83	96.83	96.83																
Brachylaima virginianum	DQ060330	96.12	96.2	96.2	96.2	97.93															
Brachylaima thompsoni	AF18262	97.85	98.08	98.08	98.08	96.8	95.95														
AR-Sporo-1	MN817937	96.52	96.6	96.6	96.6	96.2	95.13	96.73													
Brachylaima asakawai	LC349000	98.74	98.81	98.81	98.81	97.23	96.53	98.48	96.76												
Brachylaima asakawai	LC349001	98.74	98.81	98.81	98.81	100	96.53	98.48	96.76	100											
Leuchloridium paradoxum	KP938187	88.76	89.31	89.31	89.31	88.57	87.76	88.41	87.97	89.15	89.15										
Leucochloridium perturbatum	AF184261	88.65	89.21	89.21	89.21	88.46	87.76	88.41	87.85	89.05	89.05	100									
Leucochloridium perturbatum	AY222169	88.76	89.31	89.31	89.31	88.57	87.76	88.41	87.97	89.15	89.15	100	100								
Leucochloridium perturbatum	KP938186	88.76	89.31	89.31	89.31	88.57	87.76	88.41	87.97	89.15	89.15	100	100	100							
Postharmostomum commutatum	MH915390	85.33	85.66	85.66	85.66	85.45	84.95	85.66	84.83	85.57	85.57	85.07	85.07	85.07	85.07						
Urogonimus macrostomus	AB494468	89.87	90.11	90.11	90.11	89.69	88.93	90.26	89	90.27	90.27	91.46	91.37	91.46	91.46	88.79					
Urogonimus macrostomus	AY222168	89.56	89.79	89.79	89.79	89.37	88.6	89.94	88.69	89.95	89.95	91.46	91.37	91.46	91.46	88.79	99.68				
Zeylanurotrema spearei	AY222170	86.43	86.35	86.35	86.35	85.84	84.99	86.06	85.56	86.27	86.27	85.86	85.73	85.86	85.86	84.31	88.97	88.97			
Clinostomum phalacrocoracis	FJ609423	83.31	83.39	83.39	83.39	83.03	82.48	83.15	82.75	83.47	83.47	82.66	82.49	82.66	82.66	81.28	84.34	84.18	83.17		
Clinostomum cutaneum	GQ339114	83.31	83.39	83.39	83.39	83.03	82.48	83.15	82.75	83.47	83.47	82.66	82.49	82.66	82.66	81.28	84.34	84.18	83.17	99.92	
Clinostomum complanatum	MH491531	83.07	83.15	83.15	83.15	82.79	82.14	82.91	82.51	83.23	83.23	82.42	82.25	82.42	82.42	81.03	84.26	84.1	83.09	99.6	99.52

Supplementary Table 2. Percentages of identity between the COI sequences used in the present study. Values were estimated based on aligned sequences using the SeqinR package (Charif and Lobry 2007, doi:10.1007/978-3-540-35306-5_10)

Species/sample name	GenBank acc.n°	MN849923	MN848408	KT074962	MN848409	MN848411	MN848410	KT074963	KP903684	LC198314	LC198315	LC198316	LC349006	LC349007	LC349008	MK882507	MK882508	MK882506	KP903654	KP903656	KP903667	KP903644	MK882503	MK882504	MK882502	MH919409
TF-Meta-1	MN849923																									
AR-Sporo-1	MN848408	98.51																								
Brachylaima mesostoma	KT074962	84.6	86.13																							
AR-Sporo-2	MN848409	85.2	85.77	97.63																						
AR-Sporo-4	MN848411	85.5	86.03	97.8	99.62																					
AR-Sporo-3	MN848410	85.5	86.03	97.8	99.62	100																				
Brachylaima mesostoma	KT074963	89.57	87.01	96.3	98.14	98.14	98.14																			
Brachylaima mesostoma	KP903684	89.3	86.87	95.68	98.16	98.16	98.16	99.45																		
Brachylaima ezohelicis	LC198314	84.3	85.75	84.43	84.62	84.49	84.49	85.98	85.54																	
Brachylaima ezohelicis	LC198315	84.16	85.75	84.43	84.74	84.62	84.62	86.28	85.84	99.62																
Brachylaima ezohelicis	LC198316	84.3	85.88	84.43	84.87	84.74	84.74	86.59	86.14	99.62	99.75															
Brachylaima asakawai	LC349006	86.4	88.04	84.94	85.26	85.26	85.26	86.28	85.84	86.13	85.88	86.01														
Brachylaima asakawai	LC349007	86.7	88.3	85.28	85.64	85.64	85.64	86.59	86.14	86.26	86.01	86.13	99.62													
Brachylaima asakawai	LC349008	86.7	88.3	85.11	85.51	85.51	85.51	86.59	86.14	86.26	86.01	86.13	99.49	99.87												
Urogonimus macrostomus	MK882507	74.66	74.78	73.1	75	75	75	75.07	75.07	74.26	73.96	74.26	76.33	76.04	76.04											
Urogonimus macrostomus	MK882508	74.21	74.49	72.41	74.7	74.7	74.7	75.07	74.79	74.56	74.26	74.56	76.63	76.33	76.33	99.19										
Urogonimus macrostomus	MK882506	76.75	77.56	77.44	76.73	76.73	76.73	76.08	76.07	76.45	76.18	75.9	77.56	77.29	77.29	96.14	96.14									
Leucochloridium vogtianum	KP903654	78.39	78.71	76.4	76.98	76.98	76.98	75.58	76.02	77.72	77.97	77.72	77.72	77.48	77.48	74.89	74.45	76.53								
Leucochloridium vogtianum	KP903656	78.39	78.71	76.4	76.98	76.98	76.98	75.58	76.02	77.72	77.97	77.72	77.72	77.48	77.48	74.89	74.45	76.53	100							
Leucochloridium vogtianum	KP903667	78.39	78.71	76.4	76.98	76.98	76.98	75.58	76.02	77.72	77.97	77.72	77.72	77.48	77.48	74.89	74.45	76.53	100	100						
Leucochloridium perturbatum	KP903644	76.75	77.06	73.03	77.58	77.58	77.58	78.02	77.98	77.94	77.35	77.94	78.24	77.94	77.94	78.98	79.28	78.62	83.33	83.33	83.33					
Leuchloridium paradoxum	MK882503	75.79	78.93	71.05	78.57	78.57	78.57	78.93	78.93	78.21	78.21	77.86	80	80.36	80.36	78.57	78.57	79.29	83.16	83.16	83.16	86.43				
Leuchloridium paradoxum	MK882504	75.26	78.57	70.18	78.21	78.21	78.21	78.57	78.57	77.86	77.86	77.5	80	80.36	80.36	78.57	78.57	79.29	82.65	82.65	82.65	86.43	99.64			
Leuchloridium paradoxum	MK882502	76.84	79.64	71.93	79.29	79.29	79.29	79.64	79.64	78.21	78.21	77.86	80.71	81.07	81.07	79.29	79.29	80	83.67	83.67	83.67	86.43	99.29	98.93		
Postharmostomum commutatum	MH919409	79.37	80.03	77.89	78.45	78.45	78.45	78.66	78.31	79.13	78.87	79	79.13	79.26	79.26	79.29	78.99	80.89	75.99	75.99	75.99	76.47	77.5	77.5	78.21	
Clinostomum cutaneum	KP110516	46.41	47.23	44.41	46.56	46.56	46.56	47.92	47.6	47.45	47.67	47.89	48.34	48.34	48.34	47.99	47.32	45.13	45.4	45.4	45.4	47.54	47.19	47.19	47.57	46.12

Supplementary Table 3. Percentages of identity between the COI+28S supergene alignment sequences used in the present study. GenBank accession numbers refer respectively to 28S and COI sequences. Values were estimated based on aligned sequences using the SeqinR package (Charif and Lobry 2007, doi:10.1007/978-3-540-35306-5_10)

Species/sample name	GenBank acc.n°	MN817937/ MN848408	MN817938/ MN848409	MN817939/ MN848410	MN817940/ MN848411	LC349000/ LC349006	LC198310/ LC198316	MH915390/ MH919409	KP938187/ MK882502	KP938186/ KP903644	AY222168/ MK882507
AR-Sporo-1	MN817937/MN848408										
AR-Sporo-2	MN817938/MN848409	92.48									
AR-Sporo-3	MN817939/MN848410	92.58	99.85								
AR-Sporo-4	MN817940/MN848411	92.58	99.85	100							
Brachylaima asakawai	LC349000/LC349006	93.38	93.6	93.6	93.6						
Brachylaima ezohelicis	LC198310/LC198316	92.41	93.21	93.16	93.16	93.82					
Postharmostomum commutatum	MH915390/MH919409	82.92	82.72	82.72	82.72	82.92	82.72				
Leuchloridium paradoxum	KP938187/MK882502	86.35	87.32	87.32	87.32	87.45	86.68	83.6			
Leucochloridium perturbatum	KP938186/KP903644	85.55	86.67	86.67	86.67	86.67	86.36	83.01	97.54		
Urogonimus macrostomus	AY222168/MK882507	85.63	86.55	86.55	86.55	86.92	86.23	86.54	89.26	88.87	
Clinostomum cutaneum	GQ339114/KP110516	70.38	70.49	70.38	70.38	71.14	71.41	68.84	77.17	76.59	78.23
